# Noise annoyance and risk of prevalent and incident atrial fibrillation–A sex-specific analysis

**DOI:** 10.3389/fpubh.2022.1061328

**Published:** 2022-12-02

**Authors:** Omar Hahad, Manfred E. Beutel, Donya A. Gilan, Julian Chalabi, Alexander K. Schuster, Emilio Gianicolo, Karl J. Lackner, Klaus Lieb, Peter R. Galle, Philipp S. Wild, Andreas Daiber, Thomas Münzel

**Affiliations:** ^1^Department of Cardiology–Cardiology I, University Medical Center of the Johannes Gutenberg-University Mainz, Mainz, Germany; ^2^German Center for Cardiovascular Research (DZHK), Partner Site Rhine-Main, Mainz, Germany; ^3^Leibniz Institute for Resilience Research (LIR), Mainz, Germany; ^4^Department of Psychosomatic Medicine and Psychotherapy, University Medical Center of the Johannes Gutenberg-University Mainz, Mainz, Germany; ^5^Department of Psychiatry and Psychotherapy, University Medical Center of the Johannes Gutenberg-University Mainz, Mainz, Germany; ^6^Preventive Cardiology and Preventive Medicine, Department of Cardiology, University Medical Center of the Johannes Gutenberg-University Mainz, Mainz, Germany; ^7^Department of Ophthalmology, University Medical Center of the Johannes Gutenberg-University Mainz, Mainz, Germany; ^8^Institute of Medical Biostatistics, Epidemiology and Informatics (IMBEI), University Medical Center of the Johannes Gutenberg University of Mainz, Mainz, Germany; ^9^Institute of Clinical Physiology, National Research Council, Lecce, Italy; ^10^Institute of Clinical Chemistry and Laboratory Medicine, University Medical Center of the Johannes Gutenberg-University Mainz, Mainz, Germany; ^11^Department of Internal Medicine, Gastroenterology and Hepatology, University Medical Center of the Johannes Gutenberg-University Mainz, Mainz, Germany; ^12^Center for Thrombosis and Hemostasis, University Medical Center of the Johannes Gutenberg-University Mainz, Mainz, Germany; ^13^Institute of Molecular Biology (IMB), Mainz, Germany

**Keywords:** noise annoyance, atrial fibrillation, sex-specific, cardiovascular disease, environmental risk factor

## Abstract

**Background:**

While chronic exposure to high levels of noise was demonstrated to increase the risk of various cardiovascular diseases, the association between noise annoyance and risk of cardiovascular disease remains still inconsistent. Recently, we showed that noise annoyance is associated with prevalent atrial fibrillation in the general population. However, the association between noise annoyance and risk of incident atrial fibrillation as well as potential sex-differences remain still elusive.

**Methods and results:**

15,010 subjects from a German population-based cohort were examined at baseline (2007 to 2012) and follow-up five years later (2012 to 2017) to investigative the association between noise annoyance due to multiple sources and prevalent and incident atrial fibrillation. After multivariable adjustment, the results from logistic regression analyses revealed overall consistent and positive associations between noise annoyance and prevalent and incident atrial fibrillation in men, whereas this association was weaker in women, in particular with respect to incident atrial fibrillation. For instance, industrial noise annoyance was associated with 21% (95% confidence interval (CI) 9–34%) and 18% (8–29%) higher odds of prevalent atrial fibrillation in men and women, respectively. In prospective analysis, this association remained stable in men (odds ratio (OR) 1.25, 1.07–1.44), while in women no association was observed (OR 1.03, 0.89–1.18).

**Conclusions:**

The findings suggest that noise annoyance can increase the risk of incident atrial fibrillation in a large population-based cohort and that men may be more sensitive to the adverse effects of noise annoyance with regard to the risk of atrial fibrillation.

## Introduction

Environmental noise exposure, in particular due to traffic sources, is increasingly being recognized as a major public health challenge and risk factor for various diseases including cardiovascular and neuropsychiatric diseases (1–3). Over the last years, several high-quality studies have emerged to support the notion that chronic exposure to higher levels of traffic noise is associated with increased risk of various cardiovascular disease phenotypes including arterial hypertension, atrial fibrillation, ischemic heart disease, stroke, and myocardial infarction (for review see (4, 5). Previously, we have also demonstrated that the degree of noise annoyance, the subjective stress-related response to a noise stimulus, due to various sources is related to the prevalence of atrial fibrillation (6). Moreover, we could demonstrate that noise annoyance is related to higher levels of MR-proANP, a marker that reflects vascular endothelial activation, which was in turn associated with an increased risk of incident cardiovascular disease, atrial fibrillation, and all-cause mortality (7). This may suggest that in addition to the physical level of the noise stimulus, also the cognitive-emotional perception as annoying serves as an indicator of cardiovascular risk.

This is further supported by the so far only existing meta-analysis from Ndrepepa and Twardella on the relationship between noise annoyance from road traffic noise and cardiovascular disease, demonstrating an increased risk of arterial hypertension and a positive, but insignificant, association with risk of ischemic heart disease (8). Conversely, a more recent study by Pitchika et al. found no conclusive evidence for a relationship between noise annoyance and prevalent hypertension and blood pressure in in 2,552 German subjects (9). In 6,105 residents of ten European airports from the HYENA and DEBATS studies, Baudin et al. established a significant association between aircraft noise annoyance and hypertension risk (relative risk (RR) 1.06, 95% confidence interval (CI) 1.00–1.13 for highly annoyed people compared to those who were not highly annoyed) (10). Also, Baudin et al. found aircraft noise annoyance to be associated with a fair/poor self-rated health status in men living around three French airports (11).

Importantly, it remains also unclear whether noise annoyance-induced cardiovascular consequences follow a sex-specific pattern. In general, there is no conclusive evidence that allows an overall evaluation of whether noise exposure leads to more pronounced cardiovascular effects in men or women or if there are no sex-differences in cardiovascular risk at all. Thus, the aim of the present study was 1) to determine whether noise annoyance due to different sources is associated with prevalent and incident atrial fibrillation in a large population-based cohort of men and women and if so 2) whether there are sex-specific differences in noise annoyance-induced risk of atrial fibrillation.

## Methods

### Study design and sample

Data from the Gutenberg Health Study (GHS) were used for the present analysis. Comprehensive information on the study design and details were published previously (12–14). Briefly, 15,010 individuals (aged 35 to 74 years) underwent a standardized 5-h-long baseline-examination performed from 2007 to 2012 at the study center at the University Medical Center Mainz, Germany. These examinations included a variety of interviews and clinical examinations conducted in compliance with standard operating procedures. The follow-up examinations took place after 5 years of enrollment, i.e., from 2012 to 2017. All procedures conducted in the GHS were approved by the ethics committee of the Statutory Physician Board of the State Rhineland-Palatinate [reference number 837.020.07(5555)] and the local data safety commissioners and were in line with the ethical principles for medical research involving human subjects as outlined in the Declaration of Helsinki. Before inclusion of participants written informed consent was obtained. Further information on the GHS can be found in the Supplementary material (section Gutenberg Health Study).

### Noise annoyance

Self-reported noise annoyance was measured at baseline in a standardized and validated fashion as reported recently (6, 15). On the basis of a 5-point Likert scale ranging from “not at all,” over “slightly,” “moderately,” and “strongly” to “extremely,” subjects were asked to rate “how annoyed have you been in the past years by … during the day/in your sleep?”. Multiple sources of annoyance including road traffic, aircraft, railway, industrial, and neighborhood noise were assessed. Overall noise annoyance was defined as highest annoyance rating regardless of the specific noise source and of whether it affected daytime or sleep. Source-specific overall noise annoyance was defined as highest source-specific annoyance rating regardless of whether it affected daytime or sleep.

### Atrial fibrillation

Prevalent and incident atrial fibrillation was defined as either self-reported previous physician diagnosis of atrial fibrillation and/or diagnosis of atrial fibrillation on the study electrocardiogram during the baseline and follow-up examinations at the study center. Cardiac rhythm analysis was performed automatically (GE Healthcare, CardioSoft v6) and confirmed by at least two cardiologists. Electrocardiogram-based diagnosis of atrial fibrillation was defined as irregular R peak intervals and an absence of P waves. Further methodological details have been described elsewhere (16).

### Definition of covariates

Information concerning sociodemographic variables, traditional cardiovascular risk factors, and drug intake from the 5-h long baseline examination were used to provide a comprehensive statistical adjustment strategy. Detailed definitions of the covariates used in the present study can be found in the Supplementary Table S1.

### Statistical analysis

All analyses were done sex-specifically. Baseline characteristics of the study sample are shown as mean and standard deviation for continuous variables and sex differences were tested with *T*-test. Binary variables are described as relative and absolute frequencies and tested with chi-squared test. Logistic regression analysis with corresponding odds ratios (OR), 95% CI, and *p*-values were used to determine the relationship between noise annoyance and prevalent and incident atrial fibrillation. Noise annoyance was treated as a continuous variable in all models. The incident analysis was only conducted in those subjects without atrial fibrillation at baseline. Statistical analysis included sequential adjustment. Model 1 was adjusted for age (continuous). Model 2 was additionally adjusted for socioeconomic status (continuous), physical activity (continuous), alcohol consumption (binary), diabetes mellitus (binary), arterial hypertension (binary), current smoking (binary), obesity (binary), dyslipidemia (binary), family history of myocardial infarction or stroke (binary). Model 3 was additionally adjusted for medication use (antihypertensives, diuretics, beta-blockers, calcium channel blocker, agents acting on the renin-angiotensin-aldosterone system, and lipid modifying agents, all binary). In the present analysis, *p*-values should be treated as a continuous measure of statistical strength of an association, and they are therefore reported exactly. For descriptive reasons, *p*-values < 0.05 were regarded as important associations. The statistical data analyses were performed using the software R (http://www.r-project.org/).

## Results

### Baseline study sample characteristics

**Figure 1:**
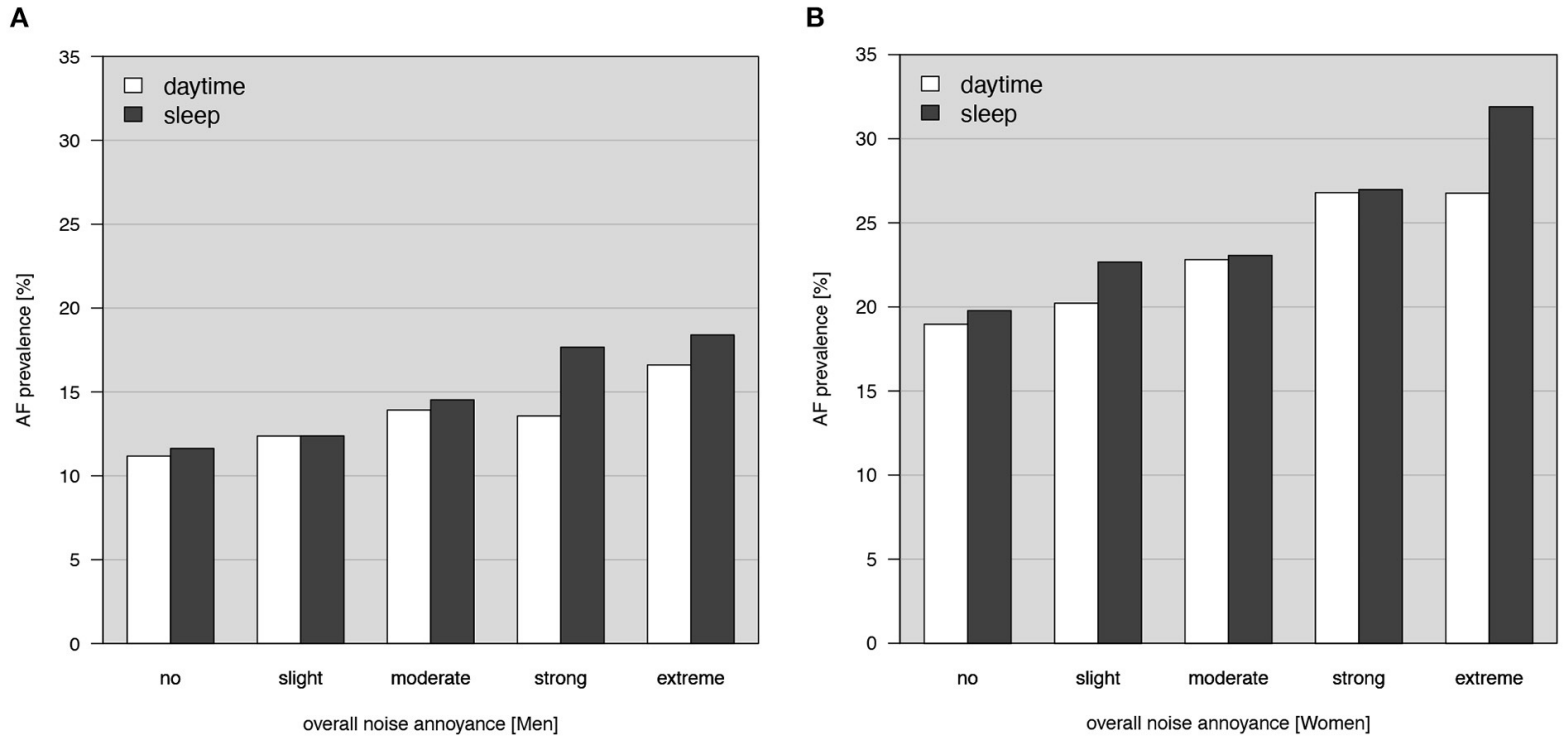
Prevalence of atrial fibrillation (AF) in relation to the degree of overall noise annoyance stratified by daytime or sleep in **(A)** men and **(B)** women.

Table 1 gives an overview of the baseline characteristics of the study sample stratified by sex. Men were older, had higher socioeconomic status, consumed more alcohol above recommended limit, whereas no differences were observed regarding physical activity compared to women. While women had in general more favorable cardiovascular risk factor and medication profile, the prevalence of atrial fibrillation was higher in women (22.6%) compared to men (13.3%). Concerning noise annoyance during the day, aircraft noise was the most prominent source affecting 60.7% of men and 56.0% of women. In agreement, aircraft noise was the biggest source of annoyance during sleep with 32.9% of men being affected and 30.1% of women. The following number of atrial fibrillation cases were identified by the respective method: 2,276 cases by electrocardiogram, 215 cases by self-reported physician diagnosis, and 192 cases by both methods. There was an increase in the prevalence of atrial fibrillation in relation to the degree of overall noise annoyance during the day and sleep in both men and women (Figure 1).

**Table 1:** Baseline characteristics of the study sample stratified by sex (*N* = 15,010).

	**Men**	**Women**	***P*-value**
	**(*n* = 7,584)**	**(*n* = 7,426)**	
Age–years	55.3 ± 11.1	54.8 ± 11.1	**0.0057**
Socioeconomic status (SES)–score	13.59 ± 4.62	12.16 ± 4.21	**< 0.0001**
Physical activity (SQUASH)–score	7.38 ± 4.32	7.35 ± 3.61	0.71
Alcohol consumption above recommended limit–no (%)	24.9 (1,888)	19.9 (1,476)	**< 0.0001**
Atrial fibrillation	13.3 (1,006)	22.6 (1,677)	**< 0.0001**
**Traditional cardiovascular risk factors**			
Current smoking–no (%)	20.8 (1,576)	18.0 (1,335)	**< 0.0001**
Diabetes mellitus–no (%)	11.4 (863)	7.2 (532)	**< 0.0001**
Hypertension–no (%)	54.6 (4,142)	44.8 (3,324)	**< 0.0001**
Obesity–no (%)	26.3 (1,991)	24.1 (1,792)	**0.0028**
Dyslipidemia–no (%)	43.1 (3,257)	25.9 (1,919)	**< 0.0001**
Family history of myocardial infarction or stroke–no (%)	20.2 (1,532)	24.1 (1,789)	**< 0.0001**
**Cardiovascular medication**			
Antihypertensives (C02)	1.1 (83)	1.0 (72)	0.47
Diuretics (C03)	5.2 (393)	5.4 (397)	0.71
Beta-blockers (C07)	17.5 (1,313)	16.6 (1,224)	0.13
Calcium channel blocker (C08)	8.3 (620)	6.4 (471)	**< 0.0001**
Agents acting on the renin-angiotensin-aldosterone system (C09)	27.4 (2,054)	20.2 (1,489)	**< 0.0001**
Lipid modifying agents (C10)	15.7 (1,175)	11.0 (809)	**< 0.0001**
**Noise annoyance**			
Road traffic noise annoyance (>0, day)–no (%)	42.3 (3,132)	40.1 (2,903)	**0.0084**
Aircraft noise annoyance (>0, day)–no (%)	60.7 (4,492)	56.0 (4,052)	**< 0.0001**
Railway noise annoyance (>0, day)–no (%)	15.5 (1,148)	13.5 (975)	**0.00051**
Industrial noise annoyance (>0, day)–no (%)	14.3 (1,055)	12.6 (912)	**0.0039**
Neighborhood noise annoyance (>0, day)–no (%)	36.3 (2,684)	35.7 (2,579)	0.46
Road traffic noise annoyance (>0, sleep)–no (%)	16.2 (1,198)	16.5 (1,192)	0.62
Aircraft noise annoyance (>0, sleep)–no (%)	32.9 (2,429)	30.1 (2,170)	**0.00028**
Railway noise annoyance (>0, sleep)–no (%)	8.7 (642)	7.4 (536)	**0.0052**
Industrial noise annoyance (>0, sleep)–no (%)	3.0 (225)	2.2 (156)	**0.00087**
Neighborhood noise annoyance (>0, sleep)–no (%)	15.4 (1,139)	17.1 (1,234)	**0.0062**

Continuous variables are shown as mean and standard deviation and tested with T-test. Binary variables are described as relative and absolute frequencies and tested with chi-squared test.

Socioeconomic status score ranges from 3 to 21 with higher values indicating higher status.

Physical activity score was calculated by multiplying total minutes of activity by the intensity score displayed per 1000-units with higher values indicating higher physical activity.

Alcohol consumption above recommended limit denotes >24 g per day for men and >12 g per day for women.

Medication is labeled with the anatomical therapeutic chemical-code. Statistically significant *P* values (*P* < 0.05) are given in bold.

### Association between source-specific overall noise annoyance and prevalent atrial fibrillation

Table 2 shows the results of the logistic regression analysis concerning the association between source-specific overall noise annoyance and prevalent atrial fibrillation in men and women. In general, consistent positive associations between annoyance due to different noise sources and risk of prevalent atrial fibrillation were observed in both men and women. The highest effect estimate in men was observed in response to industrial noise annoyance with an OR of 1.21 (95% CI 1.09–1.34) after multivariable adjustment, while in women neighborhood noise annoyance resulted in an increased risk of 22% (OR 1.22, 95% CI 1.15–1.29). The results of the cross-sectional association analysis between source-specific noise annoyance during the day/sleep and atrial fibrillation in men and women can be found in the Supplementary Table S2.

**Table 2:** Cross-sectional association analysis between source-specific overall noise annoyance and atrial fibrillation in men and women (data from the Gutenberg Health Study 2007–2012).

**Overall noise annoyance**	***N***	**Model 1**		**Model 2**		**Model 3**	
		**OR per point**	***P*-value**	**OR per point**	***P*-value**	**OR per point**	***P*-value**
		**increase [95% CI]**		**increase [95% CI]**		**increase [95% CI]**	
**Men**							
Road traffic	6,086	1.08 [1.01; 1.16]	**0.028**	1.09 [1.01; 1.17]	**0.034**	1.08 [1.00; 1.17]	0.053
Aircraft	6,086	1.06 [1.00; 1.12]	**0.034**	1.09 [1.03; 1.16]	**0.0035**	1.10 [1.03; 1.17]	**0.0026**
Railway	6,081	1.14 [1.04; 1.25]	**0.0042**	1.15 [1.04; 1.27]	**0.0066**	1.15 [1.04; 1.28]	**0.0072**
Industrial	6,082	1.15 [1.05; 1.26]	**0.0035**	1.20 [1.08; 1.33]	**0.00063**	1.21 [1.09; 1.34]	**0.00035**
Neighborhood	6,083	1.18 [1.10; 1.26]	**< 0.0001**	1.16 [1.07; 1.26]	**0.00042**	1.14 [1.04; 1.23]	**0.0026**
**Women**							
Road traffic	5,590	1.12 [1.07; 1.19]	**< 0.0001**	1.10 [1.03; 1.17]	**0.0022**	1.10 [1.04; 1.17]	**0.0018**
Aircraft	5,590	1.07 [1.02; 1.11]	**0.0036**	1.07 [1.01; 1.12]	**0.013**	1.06 [1.01; 1.12]	**0.017**
Railway	5,588	1.06 [0.98; 1.15]	0.15	1.07 [0.97; 1.17]	0.17	1.08 [0.98; 1.18]	0.097
Industrial	5,588	1.15 [1.07; 1.24]	**0.00021**	1.17 [1.08; 1.28]	**0.00024**	1.18 [1.08; 1.29]	**0.00013**
Neighborhood	5,588	1.22 [1.16; 1.28]	**< 0.0001**	1.22 [1.15; 1.30]	**< 0.0001**	1.22 [1.15; 1.29]	**< 0.0001**

Odds ratios (OR) and 95% confidence intervals (CI) are derived from a logistic regression model modeling for prevalent atrial fibrillation (dependent variable) per point increase in source-specific overall noise annoyance (independent variable). N denotes model 3.

Model 1 was adjusted for age.

Model 2 was additionally adjusted for socioeconomic status, physical activity, alcohol consumption, diabetes mellitus, arterial hypertension, current smoking, obesity, dyslipidemia, family history of myocardial infarction or stroke.

Model 3 was additionally adjusted for medication use (antihypertensives, diuretics, beta-blockers, calcium channel blocker, agents acting on the renin-angiotensin-aldosterone system, and lipid modifying agents). Statistically significant *P* values (*P* < 0.05) are given in bold.

### Association between source-specific overall noise annoyance and incident atrial fibrillation

Table 3 displays the effect estimates obtained in the incident analyses. In men, a 25% (OR 1.25, 95% CI 1.07–1.44) higher risk of incident atrial fibrillation in response to industrial noise annoyance was observed. Furthermore, road traffic (OR 1.14, 95% CI 1.01–1.28) and neighborhood noise annoyance (OR 1.19, 95% CI 1.05–1.34) independently increased the risk of incident atrial fibrillation in men. In contrast, effect estimates in women were weaker and of lower magnitude. The results of the prospective association analysis between source-specific noise annoyance during the day/sleep and atrial fibrillation in men and women can be found in the Supplementary Table S3.

**Table 3:** Prospective association analysis between source-specific overall noise annoyance and incidence of atrial fibrillation in men and women (data from the Gutenberg Health Study 2007–2017).

**Overall noise annoyance**	***N***	**Model 1**		**Model 2**		**Model 3**	
		**OR per point**	***P*-value**	**OR per point**	***P*-value**	**OR per point**	***P*-value**
		**increase [95% CI]**		**increase [95% CI]**		**increase [95% CI]**	
**Men**							
Road traffic	4,568	1.14 [1.03; 1.26]	**0.011**	1.14 [1.02; 1.28]	**0.021**	1.14 [1.01; 1.28]	**0.025**
Aircraft	4,568	1.06 [0.98; 1.15]	0.16	1.04 [0.95; 1.14]	0.38	1.05 [0.95; 1.15]	0.33
Railway	4,565	1.10 [0.95; 1.27]	0.18	1.15 [0.98; 1.33]	0.081	1.15 [0.97; 1.33]	0.084
Industrial	4,566	1.18 [1.02; 1.35]	**0.022**	1.24 [1.06; 1.43]	**0.0044**	1.25 [1.07; 1.44]	**0.0030**
Neighborhood	4,567	1.17 [1.05; 1.30]	**0.0051**	1.17 [1.03; 1.32]	**0.011**	1.19 [1.05; 1.34]	**0.0068**
**Women**							
Road traffic	3,645	1.08 [0.99; 1.17]	0.083	1.07 [0.97; 1.17]	0.18	1.07 [0.97; 1.18]	0.16
Aircraft	3,645	1.05 [0.98; 1.12]	0.18	1.04 [0.97; 1.13]	0.28	1.04 [0.97; 1.13]	0.26
Railway	3,644	1.04 [0.92; 1.17]	0.51	1.09 [0.95; 1.24]	0.20	1.08 [0.94; 1.23]	0.25
Industrial	3,644	1.07 [0.95; 1.21]	0.24	1.04 [0.90; 1.19]	0.58	1.03 [0.89; 1.18]	0.69
Neighborhood	3,644	1.05 [0.96; 1.14]	0.24	1.08 [0.98; 1.19]	0.10	1.08 [0.98; 1.19]	0.098

Odds ratios (OR) and 95% confidence intervals (CI) are derived from a logistic regression model modeling for incident atrial fibrillation (dependent variable) per point increase in source-specific overall noise annoyance (independent variable). N denotes model 3.

Model 1 was adjusted for age.

Model 2 was additionally adjusted for socioeconomic status, physical activity, alcohol consumption, diabetes mellitus, arterial hypertension, current smoking, obesity, dyslipidemia, family history of myocardial infarction or stroke.

Model 3 was additionally adjusted for medication use (antihypertensives, diuretics, beta-blockers, calcium channel blocker, agents acting on the renin-angiotensin-aldosterone system, and lipid modifying agents). Statistically significant *P* values (*P* < 0.05) are given in bold.

### Association between overall noise annoyance and prevalent and incident atrial fibrillation

In Table 4, the results of the cross-sectional and prospective association analysis between overall noise annoyance and atrial fibrillation in men and women can be found. In men, overall noise annoyance as well as overall noise annoyance during the day and sleep was consistently and positively associated with higher risk of prevalent and incident atrial fibrillation ranging from 11 to 18%, whereas in women prevalent risk of atrial fibrillation was consistently increased but not incident risk.

**Table 4:** Cross-sectional/prospective association analysis between overall noise annoyance and prevalent/incident atrial fibrillation in men and women.

	***N***	**Model 1**		**Model 2**		**Model 3**	
		**OR per point**	***P*-value**	**OR per point**	***P*-value**	**OR per point**	***P*-value**
		**increase [95% CI]**		**increase [95% CI]**		**increase [95% CI]**	
**Men**							
**Prevalent atrial fibrillation**							
Overall noise annoyance	6,087	1.14 [1.08; 1.20]	**< 0.0001**	1.16 [1.09; 1.23]	**< 0.0001**	1.16 [1.09; 1.23]	**< 0.0001**
Overall noise annoyance day	6,086	1.10 [1.04; 1.16]	**0.00090**	1.12 [1.05; 1.19]	**0.00051**	1.12 [1.05; 1.19]	**0.00054**
Overall noise annoyance sleep	6,072	1.16 [1.10; 1.23]	**< 0.0001**	1.18 [1.11; 1.25]	**< 0.0001**	1.18 [1.11; 1.26]	**< 0.0001**
**Incident atrial fibrillation**							
Overall noise annoyance	4,569	1.15 [1.06; 1.25]	**0.0010**	1.13 [1.03; 1.24]	**0.0097**	1.14 [1.04; 1.25]	**0.0063**
Overall noise annoyance day	4,568	1.12 [1.03; 1.21]	**0.011**	1.10 [1.00; 1.20]	0.054	1.11 [1.01; 1.22]	**0.038**
Overall noise annoyance sleep	4,558	1.14 [1.05; 1.24]	**0.0011**	1.13 [1.03; 1.23]	**0.0079**	1.13 [1.03; 1.24]	**0.0075**
**Women**							
**Prevalent atrial fibrillation**							
Overall noise annoyance	5,590	1.17 [1.12; 1.22]	**< 0.0001**	1.17 [1.11; 1.22]	**< 0.0001**	1.16 [1.11; 1.22]	**< 0.0001**
Overall noise annoyance day	5,590	1.15 [1.10; 1.20]	**< 0.0001**	1.13 [1.08; 1.19]	**< 0.0001**	1.13 [1.07; 1.19]	**< 0.0001**
Overall noise annoyance sleep	5,580	1.16 [1.11; 1.21]	**< 0.0001**	1.17 [1.12; 1.23]	**< 0.0001**	1.17 [1.11; 1.23]	**< 0.0001**
**Incident atrial fibrillation**							
Overall noise annoyance	3,645	1.05 [0.98; 1.12]	0.18	1.05 [0.98; 1.13]	0.18	1.05 [0.98; 1.14]	0.17
Overall noise annoyance day	3,645	1.04 [0.97; 1.11]	0.24	1.04 [0.96; 1.12]	0.36	1.04 [0.96; 1.12]	0.33
Overall noise annoyance sleep	3,640	1.04 [0.97; 1.11]	0.26	1.05 [0.98; 1.14]	0.17	1.05 [0.98; 1.13]	0.18

Odds ratios (OR) and 95% confidence intervals (CI) are derived from a logistic regression model modeling for prevalent/incident atrial fibrillation (dependent variable) per point increase in overall noise annoyance (independent variable). N denotes model 3.

Model 1 was adjusted for age.

Model 2 was additionally adjusted for socioeconomic status, physical activity, alcohol consumption, diabetes mellitus, arterial hypertension, current smoking, obesity, dyslipidemia, family history of myocardial infarction or stroke.

Model 3 was additionally adjusted for medication use (antihypertensives, diuretics, beta-blockers, calcium channel blocker, agents acting on the renin-angiotensin-aldosterone system, and lipid modifying agents). Statistically significant *P* values (*P* < 0.05) are given in bold.

## Discussion

The present study investigated the association between noise annoyance due to multiple sources and risk of prevalent and incident atrial fibrillation with further examination of sex-specific differences in a large population-based cohort. Overall, the results demonstrated that noise annoyance was consistently and positively associated with risk of prevalent and incident atrial fibrillation in men, whereas this association was weaker in women, in particular in prospective analyses. Additionally, our results emphasize that besides traffic sources of noise annoyance (road traffic, aircraft, and railways), also non-traffic sources such as industrial and neighborhood noise annoyance can increase the risk of atrial fibrillation. Importantly, sequential adjustment for covariates displayed only marginal modification of effect estimates, which may demonstrate that noise annoyance constitutes an independent risk factor beyond traditional cardiovascular risk factors. The findings of the present study add to the evidence that noise annoyance can increase the risk of cardiovascular disease with the further notion that men may be more sensitive to the adverse effects of noise annoyance with regard to the risk of atrial fibrillation.

### Noise reaction model

The positive finding of noise annoyance to increase the risk of cardiovascular disease, in the present study specifically of atrial fibrillation, corresponds with the rationale of the noise reaction scheme put forward by Babisch (17, 18). In this context, annoyance by chronic low-level noise exposure and its interference with daily routines and importantly sleep leads to an increased state of psychological arousal that is characterized by increased stress hormone levels, blood pressure, and heart rate. This, in turn, initiates and contributes to the development and acceleration of cardiovascular risk factors such as hypertension, arrhythmia, dyslipidemia, increased blood viscosity and blood glucose, and activation of blood clotting factors, finally leading to manifest cardiovascular disease (1). This rationale is in line with numerous human studies including our own studies in which we e.g., could demonstrate that noise annoyance is dose-dependently associated with the prevalence of atrial fibrillation (6). We have also conducted a series of animal studies which revealed that in particular the perception of noise as being annoying is crucial when it comes to its adverse cardiovascular side effects by comparing exposure to white noise (continuous broad band sound exposure) vs. aircraft noise (intermittent and crescendo/soften sound exposure) at the same mean sound pressure levels (19). However, the animal data also showed that sleep phase noise exposure, due to sleep fragmentation and deprivation, is the main trigger for cardiovascular complications in exposed mice (19). Interestingly, we have also demonstrated in the GHS cohort that noise annoyance is predictive of sleep disturbance (20). Of note, when mice were exposed to 90 dB(A) for 2 h/day and 110 dB(A) for 2 h/day for 30 days they developed clear signs of depressive and anxiety-like behavior, which was associated with oxidative stress and ameliorated by administration of the antioxidant N-acetylcysteine (21). In another study, mice exposed to noise (100 dB(A) for 2 months, 5 days/week, 4 h daily) showed behavioral deficits that were partially corrected by vitamin C treatment (22). In the noise-health research field, it is widely accepted that noise annoyance is a central pathway by which noise exposure (physical dimension) exerts its detrimental health effects (23).

### Noise annoyance (subjective dimension) vs. noise exposure level (objective dimension)

On the other hand, it is important to note that noise annoyance is a heterogenous psychological construct representing the totality of negative emotions and cognitions in connection with a noise source (24, 25). Previous evidence strongly suggest that noise annoyance reactions are only partly the result of acoustic exposure and its indicators such as intensity, frequency, complexity, and duration, but are also influenced by personal, social, and situational factors including age, sex, health status, noise sensitivity, attitude toward noise, socioeconomic status, public perception, perceived stress, and coping capacity (24, 25). For instance, noise annoyance may be a proxy for specific personality traits, which could underly the observed associations with atrial fibrillation rather than noise annoyance *per se* (26). When cardiovascular disease risk in response to noise is regarded as a function of actual physical exposure, then noise annoyance might be a less suited indicator of health effects as it only shares minor variance with the physical level of noise exposure. Herein it is important to acknowledge that the evidence on the relationship between noise exposure levels and risk of cardiovascular disease is much more conclusive. In support of this, recent high-quality studies rigorously demonstrated that chronic exposure to higher traffic noise levels can increase the risk of cardiovascular disease including cardiovascular death (27), hypertension (28), atrial fibrillation (29), ischemic heart disease, myocardial infarction, and heart failure (30). Importantly, a recent meta-analysis including five studies and 3,866,986 participants found a significant association between noise exposure and the risk of atrial fibrillation (RR 1.05, 95% CI 1.02–1.09) (31). However, no data concerning noise annoyance or sex-specific differences were available in this study.

### Sex-differences in noise sensitivity

As previous study results on sex-specific differences in noise-induced cardiovascular events are inconsistent, our results, indicating at stronger negative cardiovascular effect of noise annoyance in men compared to women, only partly agree with previous evidence. In correspondence with our results, a recent study suggested that men are more sensitive to transportation noise exposure by showing that nocturnal traffic noise was associated with an increased atherothrombotic risk in male myocardial infarction patients but not in female patients (32). In contrast, in an experimental setting, low-intensity noise exposure was shown to result in higher annoyance in women compared to men (26), whereas our study shows overall higher noise annoyance (>0) in men compared to women. An explanation for women being less annoyed in the present study may include the circumstance that women have better coping capacity or strategies to reduce noise stress (e.g., closing windows or physical activity) compared to men using rather maladaptive coping strategies (e.g., tobacco and alcohol use). This would explain in part the present findings of higher cardiac burden in men compared to women in response to noise annoyance. Indeed, evidence demonstrate that women are more likely to use adaptive coping strategies in stressful situations, while men are less adaptive (33, 34). Babisch et al. suggested no sex- differences in cardiovascular risk in response to traffic noise exposure (35). In line with our results, Röösli et al. revealed that men may be more sensitive to traffic noise exposure by concluding that noise-induced sleep disturbance is more prominent among men than women and thus might be a relevant mechanism by which sex-differences can be explained (36). The authors demonstrated in men who were exposed to higher levels of traffic noise (> 55 dB) compared to men who were exposed to lower traffic noise levels (< 30 dB) that sleep duration was significantly reduced by 1.5 h. Conversely, there was no effect of higher traffic noise exposure on sleep duration in women. Sex-differences in noise sensitivity may further arise from a recent study in which we demonstrated that a significant improvement of endothelial function after train noise exposure and subsequent vitamin C intake only occurred in women, although there was no difference in case of train noise-induced impaired sleep quality and endothelial dysfunction (37). This suggests that there may be differences in mechanisms causing endothelial dysfunction between men and women. In 4,821 Swedish subjects, it was demonstrated that aircraft noise exposure increased the risk of hypertension in men **(**RR 1.21, 95% CI 1.05–1.39) but not in women (RR 0.97, 95% CI 0.83–1.13) (38). Likewise, in the HYENA study, a stronger relationship between road traffic noise exposure and hypertension risk was found in men compared to women (39). This was also confirmed in the DEBATS study in which a significant association between nocturnal aircraft noise exposure and hypertension risk was found only in men (40). Taken together, sex-specific sensitivity in the setting of noise-induced cardiovascular disease remains inconsistent and, importantly, none of these studies examined sex-differences in noise annoyance-induced cardiovascular disease. A further explanation for the observed sex-differences may be the differing fat deposition in men and women. Higher cortisol levels associated with a noise annoyance-induced activation of the hypothalamic–pituitary–adrenal axis could perhaps be more detrimental for men, which are more prone to store visceral fat in the abdominal area in comparison to women who tend to have a more gluteal-femoral adipose tissue distribution (41).

### Sex or gender?

Recently, the hypothesis from Rompel et al. was put forward that gender-differences might also have explanatory value when it comes to differences between men and women in the health effects of environmental noise exposure (42). The authors comprehensively analyzed the sex/gender-differences in noise exposure-induced hypertension and ischemic heart disease on the basis of a systematic review. The authors revealed that no effect modification by sex was found in the majority of analyzed studies. They suggested that either 1) biological sex is minor important in the setting of noise-induced health effects or 2) that also gender-related differences (social, economic, and cultural factors in society) or the combination of both sex and gender might be more appropriate to explain differences in this setting. However, this is still elusive as there are no studies to date analyzing gender-related differences in the context of health effects of environmental noise exposure. Consequently, the authors concluded that stratification of a study sample on the basis of a sex/gender variable without an underlying theoretical concept is not appropriate to identify sex-differences or susceptible groups, as differences due to sex/gender variability within the groups might be greater than between them. Future studies should make efforts to disentangle between sex- and gender-related factors in the evaluation of health effects of noise (42).

### Strengths and limitations

Strengths of the present study include the large sample size of over 15,000 participants and the comprehensive and novel evaluation of multiple sources and measures of noise annoyance during the day and sleep and its associations with prevalent and incident atrial fibrillation within the same cohort. The highly standardized assessment of sociodemographic variables, cardiovascular risk factors, and medication enabled for the adjustment of a comprehensive set of relevant covariates. However, there are also limitations underlying our study. The observational, partly cross-sectional nature of the study does not allow for causal inferences and residual confounding cannot be fully excluded. As we had no data concerning objective noise exposure indicators, we considered noise annoyance to be a valid indicator of adverse noise-induced effects. We further did not assess whether participants have moved during the follow-up period. These are potential source of misclassification, which may have interfered with the present results. Another major limitation of the study is the lack of adjustment for air pollution. Air pollution is a risk factor for atrial fibrillation (43) and may be associated with noise annoyance, at least concerning traffic and industrial sources. However, it is also important to note that previous studies have indicated that air pollution and noise exposure may act independently to increase risk of atrial fibrillation (44). Also, further efforts should be made to investigate the combined effects of multiple noise sources on outcomes of interest.

## Conclusions

Noise annoyance is major health challenge affecting large parts of the population. This prospective study demonstrates that noise annoyance is related to atrial fibrillation in both men and women, while stronger effects were observed in men, especially when it comes to the incident risk of atrial fibrillation. Overall, there is increasing evidence for an association between chronic exposure to higher levels of environmental noise and cardiovascular. However, there are still gaps in the knowledge relating both to methodological differences (e.g., a lack of longitudinal studies) and low evidence concerning some exposures (e.g., lower for railway noise) and particular outcomes (e.g., atrial fibrillation) (1). Further efforts should be made to investigate the specific role of noise annoyance and sex-differences underlying the noise-disease relationship.

## Data availability statement

The raw data supporting the conclusions of this article will be made available by the authors, without undue reservation.

## Ethics statement

The studies involving human participants were reviewed and approved by Ethics Committee of the Statutory Physician Board of the State Rhineland-Palatinate (reference number 837.020.07(5555). The patients/participants provided their written informed consent to participate in this study.

## Author contributions

OH and TM conceived, designed research, carried out experiments, and drafted the manuscript. OH and JC performed data analysis. MB, DG, AS, EG, KJL, KL, PG, PW, and AD made critical contribution to the discussion and revised the manuscript. All authors read and approved the final manuscript.

## Funding

The GHS was funded through the government of Rhineland-Palatinate (Stiftung Rheinland-Pfalz für Innovation, contract AZ 961–386261/733), the research programs Wissen schafft Zukunft and Center for Translational Vascular Biology (CTVB) of the Johannes Gutenberg-University of Mainz, and its contract with Boehringer Ingelheim and PHILIPS Medical Systems, including an unrestricted grant for the GHS and by the Foundation Heart of Mainz. The funders had no role in study design, data collection and analysis, decision to publish, or preparation of the manuscript.

## Conflict of interest

The authors declare that the research was conducted in the absence of any commercial or financial relationships that could be construed as a potential conflict of interest.

## Publisher's note

All claims expressed in this article are solely those of the authors and do not necessarily represent those of their affiliated organizations, or those of the publisher, the editors and the reviewers. Any product that may be evaluated in this article, or claim that may be made by its manufacturer, is not guaranteed or endorsed by the publisher.
