# Supplementary material for: Noise annoyance and risk of prevalent and incident atrial fibrillation–A sex-specific analysis
Source: Front Public Health. 2022 Dec 2;10:1061328. doi: 10.3389/fpubh.2022.1061328 (PMC9758722; doi:10.3389/fpubh.2022.1061328)
Supplement: Supplementary file 1 [file Data_Sheet_1.docx]

**Noise annoyance and prevalent and incident risk of atrial fibrillation – a sex-specific analysis**

**Online Supplement**

**Gutenberg Health Study (GHS)**

*Design and population*

The GHS is a population-based, prospective cohort study that was initiated and carried out monocentrically at the Department of Cardiology at the University Medical Center Mainz, Germany. Originally originated as the Gutenberg Heart Study focusing on the cardiovascular spectrum, the interdisciplinary nature of the GHS has been extended to study ocular, cancer, immune, metabolic, and mental disorders in the population. The overall aim of the GHS is to identify important determinants of health and disease in order to improve risk stratification for the individual. In order to gain fundamental insight into individual health and disease development, potential influencing factors such as lifestyle, environmental, biopsychosocial, and laboratory parameters were examined. The study area covered by the GHS includes the city of Mainz and the district of Mainz-Bingen with the associated municipalities of Rhein-Nahe, Bingen, Sprendlingen-Gensingen, Gau-Algesheim, Ingelheim, Heidesheim, Nieder-Olm, Budenheim, Bodenheim, Nierstein-Oppenheim, and Guntersblum with a total population of N = 397,796 (City of Mainz n = 196,425 and district of Mainz-Bingen n = 201,371) at the start of the study in April 2007. The defined target group were men and women aged 35 to 74 (n = 210,867). A random sample was drawn from this target group, evenly stratified for age, gender and place of residence (urban vs. rural) (n = 35,008). From this random sample, representative partial samples were in turn drawn using an identical stratification method with a final study population of n = 15,010. The random samples were drawn via the residents' registration offices. Subjects with insufficient knowledge of the German language and persons who were unable to take part in the examinations at the study center due to psychological or physical complications were excluded.

*Examinations*

As part of the highly standardized five-hour baseline examination, the subjects were invited to the study center and subjected to a comprehensive battery of tests in a defined order consisting of a computer-assisted personal interview, extensive medical-technical examinations, surveys using validated questionnaires, and biobanking. Prior to this, the subjects were instructed regarding compliance with defined preconditions (e.g. fasting period of at least eight hours) in order to be able to ensure the comparability of the examination setting. All examinations used in the GHS are subject to Standard Operating Procedures (SOP) and clearly defined work instructions, carried out by the trained and experienced staff consisting of study physicians, medical-technical assistants, and medical specialists. Overall, the baseline and recruitment phase stretched from April 2007 to April 2012. From April 2012 to April 2017, an extensive follow-up examination was carried out in the study center, which had comparable conditions and a similar range of examinations to the baseline examination. Further follow-up examinations are planned every five years. The baseline examination included the assessment of the following: anthropometry, body temperature, current weather data, physical activity, nutrition, medications, electrocardiography, echocardiography, blood pressure and pulse, sonography of neck vessels, neurocardiac regulation, occlusion pressure measurement and ankle brachial index, spirometry, flow-mediated vasodilation and arterial stiffness, volume plethysmography of finger artery, digital photo plethysmographical pulse curve analysis, carbon monoxide in alveolar air, blood count, electrolytes, renal and liver function parameters, blood fat parameters, basic and special blood coagulation parameters, cardiac enzymes, inflammatory parameters, selected vitamins and hormones, parameters of oxidative stress, basic urine tests, social demographic data, access to and use of medical care, cancer prevention, gender-related questions, full medical history, classic cardiovascular risk factors, disease-related complaints and pathologies, family medical history, children, health care behavior, hobbies smoking, passive smoking, alcohol consumption, occupational history, exposure to airborne pollutants and noise, general happiness and environmental factors, domestic environment, neurocognitive function, personality, psychiatric diseases and mental disorders, everyday duties, social integration, psycho-social stress at work, life events, visual quality of life, plasma, serum, DNA, RNA, urine, gingival sulcus swab, lacrimal fluid, autorefraction, corrected visual acuity, visual field screening, intraocular pressure, biometry, slit-lamp examination, fundus photography, optical coherence tomography, and collection of tear fluid.

**Table S1.** Definition of covariates.

| Covariate | Definition |
| --- | --- |
| Socioeconomic status (SES) – score | Socioeconomic status was assessed by a validated index score (ranging from 3 to 21), providing information about educational background, current occupation, and salary (Lampert et al., 2013^1^). |
| Physical activity (SQUASH) – score | Physical activity was measured using the validated Short QUestionnaire to ASsess Health-enhancing physical activity (SQUASH, Campbell et al., 2016^2^), which contains questions on multiple activities (commuting, leisure time, household, and activities at work and school) referring to a normal week in recent months (days per week, average time per day, and intensity) for the calculation of an index score (total minutes of activity multiplied by intensity score). |
| Alcohol consumption above recommended limit | The categories “beneath” and “above recommended limit” were used to characterize self-reported alcohol consumption (cut-offs were >24 g per day for men and >12 g per day for women). |
| Current smoking | Participants’ self-reports were dichotomized into non-smokers (combining never smokers and former smokers) and current smokers (combining occasional and frequent smokers). Current smoking comprised regular or daily smoking (at least 1 cigarette per day, 7 per week, or 1 pack per month) for at least the past 6 months. The group of non-smokers included non-daily and non-regular smokers and former smokers who had a history of smoking (regular or daily smoking) for longer than 6 months and who were no current smokers. |
| Diabetes mellitus | Diabetes mellitus was defined by any of the following: diagnosis by a physician, antidiabetic treatment, fasting blood glucose level (overnight fast of at least 8 hours) ≥126 mg/dL, non-fasting blood glucose level (less than 8 hours of fasting) ≥200 mg/dL, or HbA1c ≥6.5%. |
| Hypertension | Arterial hypertension was diagnosed by the intake of antihypertensive drugs or a mean systolic blood pressure ≥140 mmHg or diastolic blood pressure ≥90 mmHg at rest (average of 2nd and 3rd standardized measurement after 8 and 11 minutes of rest). |
| Obesity | Obesity was defined as a body mass index ≥30 kg/m^2^. |
| Dyslipidemia | Dyslipidemia was present in the case of a physician diagnosis of dyslipidemia, low-density lipoprotein cholesterol/high-density lipoprotein cholesterol ratio >3.5, or triglycerides ≥150 mg/dL. |
| Family history of myocardial infarction or stroke | A positive history of myocardial infarction or stroke was recorded in a female first-degree relative ≤65 years or in a male first-degree relative ≤60 years. |
| Cardiovascular medication | Medication history was derived from medical records and personal reports and categorized according to the Anatomical Therapeutic Chemical Classification System (ATC) including intake of antihypertensives (C02), diuretics (C03), beta-blockers (C07), calcium channel blocker (C08), agents acting on the renin-angiotensin-aldosterone system (C09), and lipid modifying agents (C10). |
| ^1^Lampert T, Kroll LE, Muters S, Stolzenberg H. [measurement of the socioeconomic status within the german health update 2009 (geda)]. Bundesgesundheitsblatt Gesundheitsforschung Gesundheitsschutz. 2013;56:131-143.  ^2^Campbell N, Gaston A, Gray C, Rush E, Maddison R, Prapavessis H. The short questionnaire to assess health-enhancing (squash) physical activity in adolescents: A validation using doubly labeled water. J Phys Act Health. 2016;13:154-158. | |

**Table S2.** Cross-sectional association analysis between source-specific noise annoyance during the day/sleep and atrial fibrillation in men and women.

| Noise annoyance | *N* | Model 1 | | Model 2 | | Model 3 | |
| --- | --- | --- | --- | --- | --- | --- | --- |
|  |  | OR per point increase [95% CI] | *P* value | OR per point increase [95% CI] | *P* value | OR per point increase [95% CI] | *P* value |
| Men | | | | | | | |
| *Day* | | | | | | | |
| Road traffic | 6,085 | 1.07 [0.99; 1.15] | 0.070 | 1.08 [1.00; 1.16] | 0.063 | 1.07 [0.99; 1.16] | 0.091 |
| Aircraft | 6,083 | 1.04 [0.98; 1.10] | 0.17 | 1.07 [1.00; 1.14] | **0.034** | 1.08 [1.01; 1.15] | **0.020** |
| Railway | 6,077 | 1.10 [0.99; 1.21] | 0.066 | 1.09 [0.97; 1.22] | 0.13 | 1.09 [0.97; 1.22] | 0.15 |
| Industrial | 6,081 | 1.13 [1.02; 1.25] | **0.014** | 1.17 [1.05; 1.30] | **0.0049** | 1.18 [1.06; 1.32] | **0.0029** |
| Neighborhood | 6,082 | 1.17 [1.08; 1.26] | **<0.0001** | 1.18 [1.08; 1.29] | **0.00018** | 1.16 [1.06; 1.26] | **0.0012** |
| *Sleep* | | | | | | | |
| Road traffic | 6,071 | 1.11 [1.00; 1.23] | **0.047** | 1.08 [0.95; 1.21] | 0.22 | 1.08 [0.95; 1.21] | 0.22 |
| Aircraft | 6,070 | 1.10 [1.04; 1.17] | **0.0015** | 1.13 [1.06; 1.21] | **0.0015** | 1.14 [1.07; 1.22] | **0.00012** |
| Railway | 6,068 | 1.19 [1.06; 1.33] | **0.0030** | 1.19 [1.04; 1.35] | **0.0090** | 1.20 [1.05; 1.36] | **0.0072** |
| Industrial | 6,068 | 1.14 [0.91; 1.39] | 0.23 | 1.24 [0.98; 1.52] | 0.056 | 1.24 [0.98; 1.52] | 0.057 |
| Neighborhood | 6,070 | 1.19 [1.08; 1.31] | **0.00025** | 1.15 [1.02; 1.28] | **0.016** | 1.13 [1.01; 1.27] | **0.030** |
| Women | | | | | | | |
| *Day* | | | | | | | |
| Road traffic | 5,590 | 1.09 [1.03; 1.15] | **0.0035** | 1.05 [0.98; 1.12] | 0.15 | 1.05 [0.98; 1.12] | 0.13 |
| Aircraft | 5,589 | 1.05 [1.00; 1.10] | **0.032** | 1.04 [0.99; 1.10] | 0.12 | 1.04 [0.99; 1.10] | 0.14 |
| Railway | 5,586 | 1.03 [0.94; 1.12] | 0.50 | 1.05 [0.95; 1.15] | 0.35 | 1.06 [0.96; 1.17] | 0.27 |
| Industrial | 5,585 | 1.16 [1.07; 1.25] | **0.00016** | 1.17 [1.07; 1.28] | **0.00036** | 1.18 [1.08; 1.29] | **0.00021** |
| Neighborhood | 5,588 | 1.23 [1.16; 1.30] | **<0.0001** | 1.22 [1.15; 1.31] | **<0.0001** | 1.22 [1.14; 1.30] | **<0.0001** |
| *Sleep* | | | | | | | |
| Road traffic | 5,578 | 1.23 [1.15; 1.33] | **<0.0001** | 1.26 [1.16; 1.37] | **<0.0001** | 1.27 [1.17; 1.38] | **<0.0001** |
| Aircraft | 5,577 | 1.10 [1.05; 1.15] | **0.00012** | 1.11 [1.05; 1.17] | **0.00028** | 1.10 [1.04; 1.16] | **0.00046** |
| Railway | 5,574 | 1.16 [1.04; 1.28] | **0.0045** | 1.13 [1.01; 1.27] | **0.035** | 1.15 [1.02; 1.30] | **0.016** |
| Industrial | 5,572 | 1.21 [1.00; 1.43] | **0.038** | 1.21 [0.98; 1.47] | 0.068 | 1.24 [1.00; 1.51] | **0.041** |
| Neighborhood | 5,576 | 1.25 [1.17; 1.33] | **<0.0001** | 1.27 [1.17; 1.36] | **<0.0001** | 1.26 [1.17; 1.36] | **<0.0001** |

Odds ratios (OR) and 95% confidence intervals (CI) are derived from a logistic regression model modeling for prevalent atrial fibrillation (dependent variable) per point increase in source-specific noise annoyance during day/sleep (independent variable). *N* denotes model 3. Statistically significant *P* values (*P* < 0.05) are given in bold.

Model 1 was adjusted for age

Model 2 was additionally adjusted for socioeconomic status, physical activity, alcohol consumption, diabetes mellitus, arterial hypertension, current smoking, obesity, dyslipidemia, family history of myocardial infarction or stroke

Model 3 was additionally adjusted for medication use (antihypertensives, diuretics, beta-blockers, calcium channel blocker, agents acting on the renin-angiotensin-aldosterone system, and lipid modifying agents)

**Table S3.** Prospective association analysis between source-specific noise annoyance during the day/sleep and atrial fibrillation in men and women.

| Noise annoyance | *N* | Model 1 | | Model 2 | | Model 3 | |
| --- | --- | --- | --- | --- | --- | --- | --- |
|  |  | OR per point increase [95% CI] | *P* value | OR per point increase [95% CI] | *P* value | OR per point increase [95% CI] | *P* value |
| Men | | | | | | | |
| *Day* | | | | | | | |
| Road traffic | 4,567 | 1.10 [0.98; 1.22] | 0.086 | 1.10 [0.97; 1.23] | 0.12 | 1.11 [0.98; 1.24] | 0.10 |
| Aircraft | 4,566 | 1.04 [0.96; 1.13] | 0.35 | 1.03 [0.93; 1.13] | 0.55 | 1.04 [0.94; 1.14] | 0.48 |
| Railway | 4,562 | 1.07 [0.90; 1.24] | 0.43 | 1.13 [0.95; 1.33] | 0.16 | 1.13 [0.95; 1.33] | 0.16 |
| Industrial | 4,565 | 1.20 [1.03; 1.37] | **0.014** | 1.26 [1.07; 1.45] | **0.0031** | 1.27 [1.09; 1.47] | **0.0020** |
| Neighborhood | 4,566 | 1.15 [1.01; 1.29] | **0.025** | 1.14 [0.99; 1.30] | 0.059 | 1.15 [1.00; 1.31] | **0.043** |
| *Sleep* | | | | | | | |
| Road traffic | 4,557 | 1.26 [1.09; 1.44] | **0.0011** | 1.31 [1.12; 1.52] | **0.00046** | 1.30 [1.11; 1.51] | **0.00070** |
| Aircraft | 4,557 | 1.09 [1.00; 1.19] | **0.049** | 1.06 [0.96; 1.17] | 0.26 | 1.06 [0.96; 1.17] | 0.25 |
| Railway | 4,555 | 1.16 [0.96; 1.38] | 0.11 | 1.20 [0.98; 1.44] | 0.062 | 1.21 [0.98; 1.45] | 0.061 |
| Industrial | 4,555 | 1.19 [0.87; 1.55] | 0.23 | 1.29 [0.48; 1.68] | 0.080 | 1.30 [0.95; 1.70] | 0.069 |
| Neighborhood | 4,557 | 1.22 [1.05; 1.40] | **0.0054** | 1.27 [1.09; 1.48] | **0.0018** | 1.29 [1.10; 1.50] | **0.0010** |
| Women | | | | | | | |
| *Day* | | | | | | | |
| Road traffic | 3,645 | 1.07 [0.98; 1.17] | 0.13 | 1.05 [0.95; 1.16] | 0.34 | 1.05 [0.95; 1.16] | 0.31 |
| Aircraft | 3,644 | 1.05 [0.98; 1.12] | 0.21 | 1.04 [0.96; 1.12] | 0.35 | 1.04 [0.96; 1.12] | 0.33 |
| Railway | 3,643 | 0.98 [0.85; 1.12] | 0.74 | 1.02 [0.87; 1.18] | 0.82 | 1.00 [0.86; 1.16] | 0.95 |
| Industrial | 3,641 | 1.08 [0.95; 1.22] | 0.22 | 1.04 [0.89; 1.19] | 0.60 | 1.03 [0.88; 1.18] | 0.71 |
| Neighborhood | 3,644 | 1.09 [0.99; 1.19] | 0.078 | 1.10 [0.99; 1.22] | 0.075 | 1.10 [0.99; 1.22] | 0.067 |
| *Sleep* | | | | | | | |
| Road traffic | 3,639 | 1.12 [0.99; 1.25] | 0.071 | 1.14 [1.00; 1.30] | **0.046** | 1.14 [1.00; 1.30] | 0.051 |
| Aircraft | 3,639 | 1.02 [0.94; 1.09] | 0.70 | 1.01 [0.92; 1.10] | 0.84 | 1.01 [0.93; 1.10] | 0.82 |
| Railway | 3,639 | 1.14 [0.97; 1.33] | 0.097 | 1.21 [1.02; 1.43] | **0.022** | 1.20 [1.00; 1.41] | **0.036** |
| Industrial | 3,637 | 1.10 [0.81; 1.44] | 0.49 | 1.09 [0.75; 1.48] | 0.62 | 1.08 [0.75; 1.47] | 0.63 |
| Neighborhood | 3,639 | 1.02 [0.90; 1.14] | 0.79 | 1.05 [0.91; 1.19] | 0.49 | 1.05 [0.92; 1.19] | 0.48 |

Odds ratios (OR) and 95% confidence intervals (CI) are derived from a logistic regression model modeling for incident atrial fibrillation (dependent variable) per point increase in source-specific noise annoyance during day/sleep (independent variable). *N* denotes model 3. Statistically significant *P* values (*P* < 0.05) are given in bold.

Model 1 was adjusted for age

Model 2 was additionally adjusted for socioeconomic status, physical activity, alcohol consumption, diabetes mellitus, arterial hypertension, current smoking, obesity, dyslipidemia, family history of myocardial infarction or stroke

Model 3 was additionally adjusted for medication use (antihypertensives, diuretics, beta-blockers, calcium channel blocker, agents acting on the renin-angiotensin-aldosterone system, and lipid modifying agents)
